# The Instant Spontaneous Neuronal Activity Modulation of Transcutaneous Auricular Vagus Nerve Stimulation on Patients With Primary Insomnia

**DOI:** 10.3389/fnins.2020.00205

**Published:** 2020-03-13

**Authors:** Bin Zhao, Yanzhi Bi, Liang Li, Jinling Zhang, Yang Hong, Lei Zhang, Jiakai He, Jiliang Fang, Peijing Rong

**Affiliations:** ^1^Institute of Acupuncture and Moxibustion, China Academy of Chinese Medical Sciences, Beijing, China; ^2^CAS Key Laboratory of Mental Health, Institute of Psychology, Beijing, China; ^3^Department of Psychology, University of Chinese Academy of Sciences, Beijing, China; ^4^Department of Radiology, Guang’anmen Hospital, China Academy of Chinese Medical Sciences, Beijing, China

**Keywords:** primary insomnia, transcutaneous auricular vagus nerve stimulation, spontaneous neuronal activity, amplitude of low frequency fluctuations, functional connectivity

## Abstract

Primary insomnia (PI) is associated with increased spontaneous neuronal activity. Transcutaneous auricular vagus nerve stimulation (taVNS) modulates brain function, and it is an effective treatment for primary insomnia. However, whether taVNS alleviates insomnia through modulating spontaneous neuronal activity is not fully clarified. This study aims to investigate the instant effect of taVNS in modulating spontaneous neuronal activity in PI patients using resting-state functional magnetic resonance imaging (rs-fMRI). Twenty-two PI subjects underwent rs-fMRI scanning prior and immediately after 30 min treatment of taVNS controlled by twenty healthy adults. Amplitude of low frequency fluctuations (ALFF) analysis was employed to assess the difference in spontaneous neuronal activity between PI patients and healthy adults, as well as between pre-treatment and post-treatment of taVNS. The taVNS-induced altered ALFF brain areas were then selected as regions of interest to perform the resting state functional connectivity (RSFC) analysis in PI patients. The right precuneus showed significantly increased ALFF in PI patients. After immediate taVNS treatment, the ALFF was significantly decreased in the right precuneus and increased in the left middle occipital gyrus. The RSFC in right precuneus with right angular, right superior frontal gyrus, and right middle frontal gyrus was significantly decreased. This study provides insights into the instant brain effects of taVNS on PI patients.

## Introduction

Insomnia is defined as a series of symptoms, such as the difficulty of falling asleep, shortened sleep duration, early waking up, sleep insufficiency or poor sleep quality (Brzezinski, 1997; Cunnington et al., 2013). Insomnia is highly prevalent, affecting approximately 10% of the adult population. In China, up to 15% of individuals are suffering from insomnia (Roehrs and Roth, 2012; Cao et al., 2017). Long-term insomnia not only disfunctions the neuroendocrine-immune interactions, but also contributes to complications such as anxiety, depression and hypertension. Currently available treatments are far from satisfactory because of their low efficacy and side effects (Duss et al., 2018). Furthermore, the underlying neural mechanisms and hereditary factors are poorly understood.

Transcutaneous auricular vagus nerve stimulation (taVNS), a non-invasive nerve electrical stimulation technique, has shown promising effectiveness on primary insomnia (PI) (Luo et al., 2017). However, the underlying mechanism of taVNS in alleviating PI is not fully clarified. A recent fMRI study illustrated that PI was associated with altered connection properties of intra-networks within the executive control network, default mode network (DMN) and salience network (Liu et al., 2018). Our previous studies showed that taVNS can alleviate insomnia in depression patients by regulating the function of DMN in depression (Fang et al., 2016). The taVNS treatment also regulated the DMN, visual relevant cortex and emotional circuits of patients suffered from insomnia after stroke (Zhao et al., 2019).

Amplitude of low frequency fluctuations (ALFF) is a widely used method in rs-fMRI data analysis, and it calculates the low frequency oscillation signal of a certain region of the brain within a period of time. The average amplitude value reflects the spontaneous activity intensity of the brain region during this period (Zang et al., 2007; Hoptman et al., 2010). Previous studies have shown that there are widely abnormal in spontaneous neuronal activity in the temporal lobe, prefrontal lobe, occipital lobe, and visual cortex-related brain regions of PI patients or sleep-deprived persons (Gao et al., 2015; Dai et al., 2016). Therefore, in this study, it was supposed that taVNS may modulate the spontaneous neuronal activity in specific brain regions on PI.

## Materials and Methods

### Participants

This study was registered as a part of the clinical trial of the PI patient treated with taVNS (clinical trial number: ChiCTR-TRC-13003519). Twenty-two participants with PI and twenty healthy participants without insomnia symptoms were included in the study. All subjects were recruited from the acupuncture and moxibustion hospital of the Chinese Academy of Chinese Medical Sciences by advertisement. The study was approved by the Ethics Committee of the Acupuncture and Moxibustion Institute of the Chinese Academy of Chinese Medical Sciences. Written informed consent was obtained from each participant before the experiment.

All PI subjects met the diagnostic criteria in the Fifth Edition of the American Diagnostic and Statistical Manual of Mental Disorders (DSM-V, 2015). Inclusion criteria: (1) 18–65 years of age, right handed; (2) the Pittsburgh Sleep Quality Index (PSQI) score ≥8 points; (3) the Hamilton Rating Scale for Depression score <17 points and the Hamilton Rating Scale for Anxiety score <14 points, excluding patients whose symptoms of depression and anxiety precede symptoms of insomnia; (5) patients stopped taking anti-insomnia medication or other psychiatric medications and receiving acupuncture treatment for over one month; (6) no history of neurological or psychiatric disorders such as depression, stroke, schizophrenia, or obsessive-compulsive disorder; (7) no contraindications (e.g., non-removable metallic implants, claustrophobia, etc.) for MRI scanning.

### Treatment Programs

The taVNS device (Hwato, SDZ-IIB, Suzhou, China) was used to apply stimulation for patients. Two surface electrodes for taVNS were pasted on each auricular concha area for both ears (Fang et al., 2016). The pulse frequency of the stimulus was adjusted to 20 Hz using density wave (pulse width: 0.2 ms ± 30%) and the intensity adjusted based on the tolerance of the patient (∼ 4–6 mA). The stimulation duration was 30 min.

### Magnetic Resonance Imaging Data Acquisition

**FIGURE 1:**
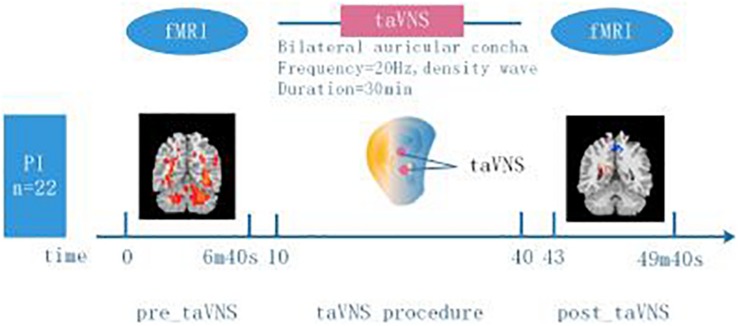
Experiment design. 22 participants with PI and 20 healthy participants without insomnia symptoms were included in the study. Two minutes and twenty seconds before (“pre_taVNS”) and three minutes after (“post_taVNS”) taVNS, resting state brain imaging data was acquired using 3.0T MRI. The taVNS stimulation lasted for 30 min delivered to bilateral auricular concha (frequency = 20 Hz, density wave). Healthy participants just underwent rs-fMRI scanning when they were recruited.

MRI data was collected on a 3.0T Siemens Skyra Scanner with the standard 20-channel head coil (Guang An Men Hospital). The subjects were asked to keep eyes closed, remain still and not to think about anything systematically. The resting-state functional images were obtained by using the echo planar imaging sequence (BOLD) with the following parameters: repetition time (TR) = 2500 ms, echo time (TE) = 30 ms, flip angle (FA) = 90°, field of view (FOV) = 240 × 240 mm, in-plane resolution = 64 × 64, slice thickness = 3.0 mm, slices = 43, slice spacing = 1.0 mm, volumes = 144. Every scan lasted 6 min and 40 s, then collected three-dimensional (3D) high-resolution full brain structural image (T1). The parameters were as the following: 176 images with sagittal orientation, slice thickness = 1.0 mm, in-plane resolution = 256 × 192, TR = 2530 ms, TE = 2.98 ms, FA = 7°, FOV = 256 × 256 mm. The PI patients underwent rs-fMRI scanning before and immediately after 30 min treatment of taVNS. As a control, the healthy participants just acquired the rs-fMRI scanning once without taVNS treatment (Experiment design Figure 1).

### Resting State Functional Imaging Data Processing

Functional data analysis was performed using Data Processing and Analysis for (Resting-State) Brain Imaging (DPABI) software^1^. Preprocessing steps included: removing the first ten time points; slice timing correction; head motion correction, the translation in the *x*, *y* or *z* direction exceeding 1.5 mm and the rotation exceeding 1.5° were eliminated (non-conformity of head movement data was rescanned); nuisance covariates regression including head motion, white matter, and cerebrospinal fluid; spatial normalization by using EPI templates (voxel size is 3 mm × 3 mm × 3 mm); spatially smoothing using a Gaussian kernel of 6mm full width at half maximum (FWHM); and filter (bandpass, 0.01–0.08 Hz) to reduce the bias from low-frequency drifts and high-frequency physiological noise.

In the present study, the ALFF analysis was conducted to examine: 1) the spontaneous neuronal activity differences between PI patients and healthy subjects, and 2) the instant regulation effects of taVNS on spontaneous neuronal activity in PI patients. Then, the brain regions showing both abnormal pre-treatment spontaneous neuronal activities and taVNS-induced spontaneous neuronal activities changes in PI patients were selected as the regions of interest (ROIs), and resting state functional connectivity (RSFC) analysis was performed to investigate the taVNS-induced brain circuit connectivity changes in PI patients.

#### Amplitude of Low Frequency Fluctuations Analysis

The filtered time series was transformed to a frequency domain using a fast Fourier transform and the power spectrum was obtained. Then the square root of the power spectrum between 0.01 and 0.08 Hz was calculated and taken as the ALFF. The ALFF of each voxel was divided by the global mean ALFF value to obtain the standardized ALFF for subsequent statistical analysis.

An independent two sample *t*-test was employed to investigate the ALFF differences between PI patients before taVNS treatment and healthy subjects. The statistical significance was set at an individual voxel probability threshold of *p* < 0.01 and a cluster size of 95 voxels, which corresponded to a corrected *p* < 0.05 (Alphasim correction). A paired sample *t*-test was performed to evaluate the taVNS treatment induced regional brain function (ALFF) changes in PI patients. Again, clusters greater than 57 voxels and an individual voxel probability threshold of *p* < 0.01 were applied to a significance level of *p* < 0.05 (Alphasim correction).

#### Resting State Functional Connectivity Analysis

Resting state functional connectivity analysis was performed in both pre-treatment and post-treatment session. The ROIs were acquired from the overlapped brain regions showing ALFF differences between those before taVNS treatment minoring the healthy control and taVNS-induced. A paired sample *t*-test was performed to investigate the taVNS treatment induced circuit changes in PI patients. The statistical significance was set at an individual voxel probability threshold of *p* < 0.01 and clusters greater than 60 voxels, which corresponded to a corrected *p* < 0.05 (Alphasim correction).

## Results

### Clinical Results: Demographic, PSQI, HAMD, and HAMA (Table 1)

The demographic and behavioral data are provided in Table 1, in which there were no significant differences in age and gender between PI patients and healthy participants. However, the PSQI, HAMD and HAMA scores were higher for the PI patients group (*n* = 22:20, *p* < 0.01 or *p* < 0.05).

**TABLE 1:** Group demographics and clinical measures.

**Measure (mean ± SD)**	**Participants with insomnia (*n* = 22)**	**Healthy participants (*n* = 20)**	***p* Value**
Age, years Sex (male/female) PSQI HAMD HAMA	42.57 ± 14.50 9/13 12.43 ± 3.50 8.57 ± 3.47 7.43 ± 3.85	36.21 ± 0.34 8/12 4.63 ± 1.38 5.52 ± 1.22 5.31 ± 1.25	0.12# 0.34Δ <0.01# <0.01# <0.05#

*SD, standard deviation; PSQI, Pittsburgh Sleep Quality Index; HAMD, Hamilton Rating Scale for Depression; HAMA, Hamilton Rating Scale for Anxiety. # Indicates *p* values for two-sample *t*-tests. Δ Indicates *p* values for chi-square test.*

### fMRI Results (1): Different ALFF in Precuneus and Occipital Gyrus (Table 2 and Figures 2A,B)

**FIGURE 2:**
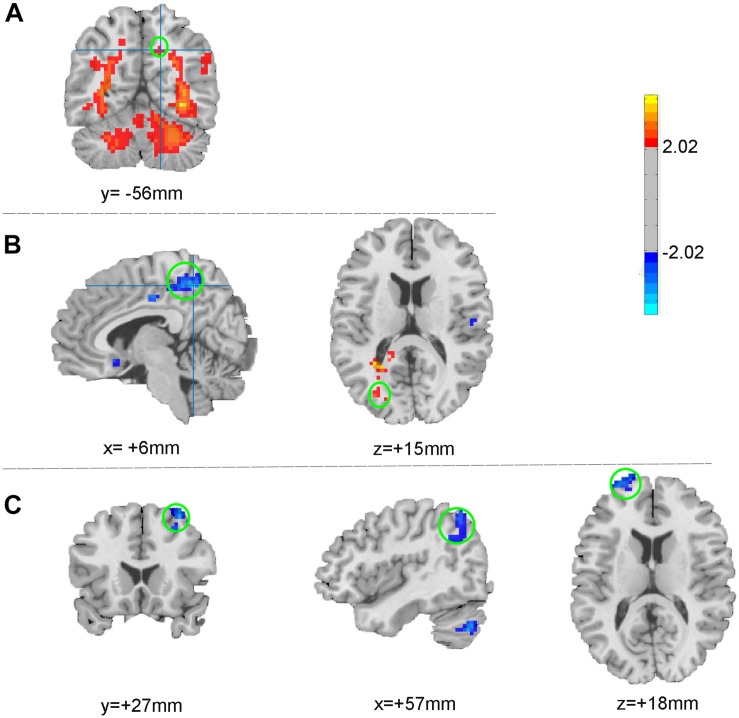
ALFF changes between patients and healthy participants **(A)** and between pre- and post- treatment **(B)**; FC changes after taVNS treatment **(C)**. The warm color indicates the regions showing increased ALFF (FC) after treatment, while the cold color indicates the opposite.

In comparison with the healthy subjects, right precuneus showed significantly increased ALFF in PI patients (Alphasim correction, Table 2 and Figure 2A). After taVNS treatment, the ALFF was significantly decreased in the right precuneus and increased in the left middle occipital gyrus in PI patients (Alphasim correction, Table 2 and Figure 2B).

**TABLE 2:** ALFF differentiation between PI and healthy participants; ALFF and FC changes with right precuneus after taVNS treatment.

**Brain regions**	**Side**	**Talairach**			**Number of**	***t* values**
		**coordinates**			**voxels**	
		**x**	**y**	**z**		
**PI > HC(Pre_ALFF)**						
Precuneus	right	16	−56	40	71	10.0
**Pre_ALFF > Post_ALFF**						
Precuneus	right	6	−48	51	55	−4.3
**Post_ALFF > Pre_ALFF**						
Middle occipital gyrus	left	−29	−76	15	77	4.4
**Pre_FC > Post_FC**						
Angular	right	57	−57	48	91	−3.4
Superior frontal gyrus	right	−27	66	18	37	−3.9
Middle frontal gyrus	right	39	27	54	49	−3.7

*ALFF, amplitude of low-frequency fluctuation; FC, functional connectivity; PI, primary insomnia; HC, healthy participants; Pre, before taVNS treatment; Post, after taVNS treatment.*

### fMRI Results(2): Decreased FC in the Right Precuneus and Right Cerebral Cortex (Table 2 and Figure 2C)

As the spontaneous neuronal activity of right precuneus showed significant differences between those before taVNS treatment minoring healthy control as well as taVNS treatment induced changes in PI patients, this region (MNI coordinates: 6, −48, 51, Radius = 6 mm) was selected as the ROI to perform RSFC analysis. After taVNS treatment, the RSFC of right precuneus with right angular, right superior frontal gyrus, and right middle frontal gyrus was significantly decreased in PI patients (Alphasim correction, Table 2 and Figure 2C).

## Discussion

In this study, we comprehensively evaluated the instant effects of taVNS on brain spontaneous neuronal activity and functional connectivity in patients with PI. Generally, the ALFF of right precuneus was significantly increased in PI patients in comparison with healthy adults. Interestingly, after 30 min of taVNS treatment on PI patients, ALFF in the right precuneus decreased, but the left middle occipital gyrus increased. Furthermore, the ROI-based RSFCs were significantly decreased between right precuneus and right angular, right superior frontal gyrus, right middle frontal gyrus.

### taVNS Eliciting the Instant Effect of Spontaneous Neuronal Activity in Precuneus

Previous neuroimaging and neurocognitive studies have confirmed that hyperarousal theory is the key mechanism for insomnia (Harvey, 2002). The theory states that because the modulation between sleep and wake-up systems is imbalanced, cortex associated with the sleep-wake cycle of PI patients is always in the awake state, which makes it challenging for the patients to fall asleep, stay asleep, and obtain refreshing sleep (Bonnet and Arand, 1995). When an individual is awake and yet not actively engaged in an attention-demanding task, a default state of brain activity exists that involves the medial prefrontal cortex and the posterior cingulate and precuneus (Raichle et al., 2001). The precuneus is mainly involved in episodic memory, emotional regulation, and introspection (Marques et al., 2018). In addition, the DMN is associated with the hyperarousal of brain regions (Marques et al., 2015). Under normal physiological conditions, the neurological activity of DMN gradually decreases after falling asleep, whereas the DMN of PI patients is still active (Horovitz et al., 2009; Sämann et al., 2011). DMN in insomnia also showed hyper-responses to arousal to sleep-related stimuli of psychophysiological insomnia, and the effective cognitive behavioral therapy (CBT) could reduce the hyper-responses (Kim et al., 2017).

Introspection of PI patients can excite DMN, which in turn affects patients’ sleep (Marques et al., 2015). Studies have shown that negative cognitive activities of PI patients who are overly concerned about their sleep quality indirectly contribute to increased brain glucose metabolic rate and brain autonomic arousal, which is one of the critical factors that cause sleep disorders (Nofzinger et al., 2004; Bonnet and Arand, 2010). The precuneus is the chief node of DMN, and the abnormality of nerve spontaneous activity is the main cause of self-response disorder in PI patients (Zhou et al., 2017). In the present study, the RSFC between the precuneus and right angular, right superior frontal gyrus, right middle frontal gyrus was significantly decreased after the taVNS treatment. Therefore, regulating the spontaneous activity of the precuneus neurons, inhibiting the patient’s introspection, and improving the hyperarousal of the cerebral cortex may be one of the key mechanisms of taVNS being beneficial on PI.

### taVNS Involving in Visual Relevant Cortical Activity

Abnormalities in visual cortical neuron function may be also one of the important factors of PI (Killgore et al., 2013; Zhao et al., 2015). The occipital lobe is not only an important region for processing and integrating the visual information, but also a major area for consolidating the visual memory process (Wang et al., 2007). Interestingly, a study has shown that sleep deprivation subjects have reduced visual information processing ability (Gao et al., 2015). However, neurons in the visually relevant cortical area are abnormally active at rest (Kong et al., 2011), which could be a compensation mechanism. Previous case reports of taVNS in the treatment of depression and post-stroke insomnia have also suggested that taVNS can modulate the function of visual relevant cortex (Li et al., 2018; Zhao et al., 2019). Therefore, taVNS may play a role of the instant effects on PI by regulating the spontaneous activity of visual relevant cortical neurons.

## Limitations

There were several limitations associated with the study. First, the current design did not enable the differentiation between taVNS and sham-taVNS in order to clarify further whether the observed abnormalities are caused by taVNS specifically. Second, the study was focused on the instant modulation of spontaneous neuronal activity in insomnia patients, long-term treatment should be included in the future research. Third, the absence of taVNS in healthy adults and multiple corrections might have limited our hypothesis. Nonetheless, the current study suggests that taVNS may play a role in the treatment of PI by modulating the spontaneous activity of neurons in the right precuneus and visual cortex-related brain regions. Further replication studies with larger sample sizes and repetitive designs are needed to confirm our findings.

## Data Availability Statement

The datasets generated for this study are available on request to the corresponding author.

## Ethics Statement

This study was carried out in the acupuncture and moxibustion hospital of the Chinese Academy of Chinese Medical Sciences. The protocol was reviewed and approved by the Acupuncture and Moxibustion Institute of the Chinese Academy of Chinese Medical Sciences Ethics Committee, and all participants provided written informed consent.

## Author Contributions

PR and JF contributed to the design of the study. BZ, YH, LZ, JZ, JF, and JH contributed to the data acquisitions. BZ, YB, JH, and JF contributed to the data analysis. BZ, JF, LL, and PR contributed to the results interpretation. BZ, YB, JF, LL, JZ, JH, and PR contributed to manuscript preparation. All authors contributed to the manuscript revision and approved the final version of the manuscript.

## Conflict of Interest

The authors declare that the research was conducted in the absence of any commercial or financial relationships that could be construed as a potential conflict of interest.

## Footnotes

1 http://rfmri.org/DPABI
